# Skull Metastasis as Initial Manifestation of Pulmonary Epithelial-Myoepithelial Carcinoma: A Case Report of an Unusual Case

**DOI:** 10.1155/2011/610383

**Published:** 2011-07-12

**Authors:** Masamitsu Nishihara, Naoya Takeda, Shoutarou Tatsumi, Keiji Kidoguchi, Shigeto Hayashi, Takashi Sasayama, Eiji Kohmura, Kimio Hashimoto

**Affiliations:** ^1^Department of Neurosurgery, Nishi-Kobe Medical Center, Nishi-ku, Kobe 651-2273, Japan; ^2^Department of Neurosurgery, Kobe University Graduate School of Medicine, Chuo-ku, Kobe 650-0017, Japan; ^3^Department of Diagnostic Pathology, Nishi-Kobe Medical Center, Nishi-ku, Kobe 651-2273, Japan

## Abstract

Epithelial-myoepithelial carcinoma (EMC) of the lung is rare and is considered to be low-grade malignancy. Intracranial metastasis of pulmonary EMC has not previously been reported according to our search of the literature. We report a case of skull metastasis as the initial manifestation of pulmonary EMC. An 81-year-old man complained of left leg motor weakness. Neurological examination showed left hemiparesis. Computed tomography and magnetic resonance imaging revealed an osteolytic tumor in the right frontal bone with invasion to the dura and subdural space, attached to the superior sagittal sinus. Subtotal removal of the tumor was performed, and the left hemiparesis showed improvement. Histopathological study revealed the tumor to consist of epithelial and myoepithelial cells. Pulmonary EMC was diagnosed. The MIB-1 index in primary lesion was approximately 10%. The skull and dura are possible sites for metastasis from pulmonary EMC. The MIB-1 index is a predictive marker of malignant potential.

## 1. Introduction

Epithelial-myoepithelial carcinoma (EMC) of the lung is rare and is considered to be low-grade malignancy. To date, approximately 20 cases have been reported in the English literature [1]. Intracranial metastasis of pulmonary EMC has not previously been reported according to our search of the literature. We report a case of skull metastasis as the initial manifestation of pulmonary EMC and discuss its malignant potential. 

## 2. Case Presentation

**Figure 1:**
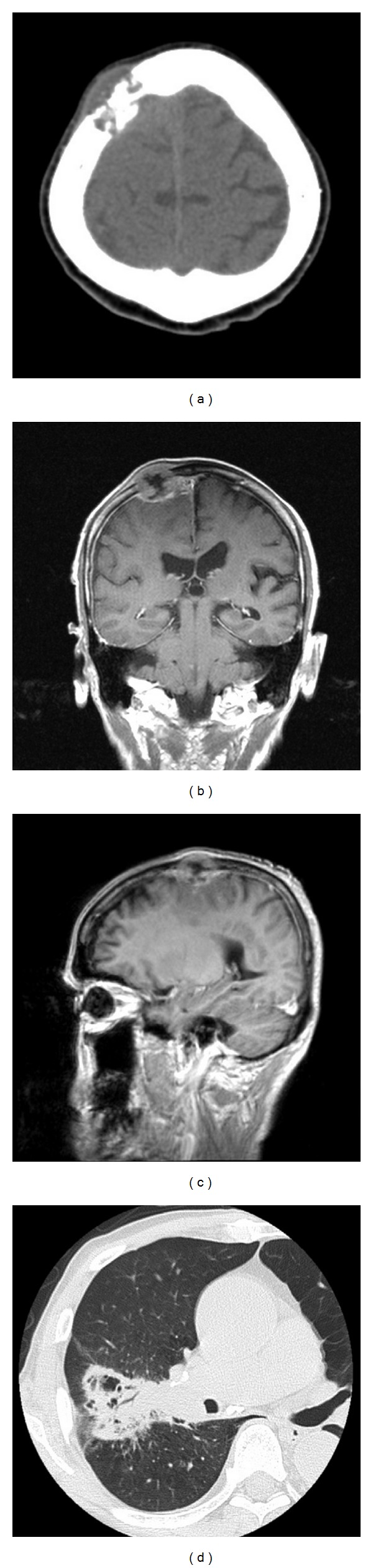
Computed tomographic scan of the brain shows an osteolytic tumor in the right frontal region (a). Coronal (b) and sagittal (c) T1-weighted magnetic resonance images with gadolinium-diethylenetriaminepenta-acetic acid revealing a heterogeneously enhanced tumor in the right frontal region with perifocal edema. The tumor was attached to the wall of the superior sagittal sinus and dura. Computed tomographic scan of the chest reveals an endobronchial growth in the hilum of the right upper lobe (d).

**Figure 2:**
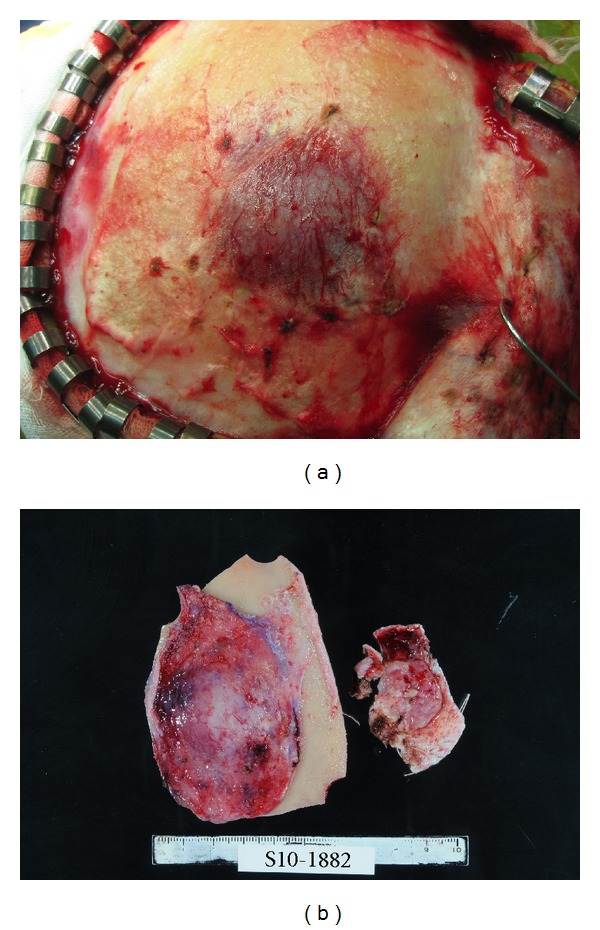
Intraoperative photographs of the tumor. (a) Complete exposure of the tumor covered by the periosteum. (b) Resected tumor with bone.

**Figure 3:**
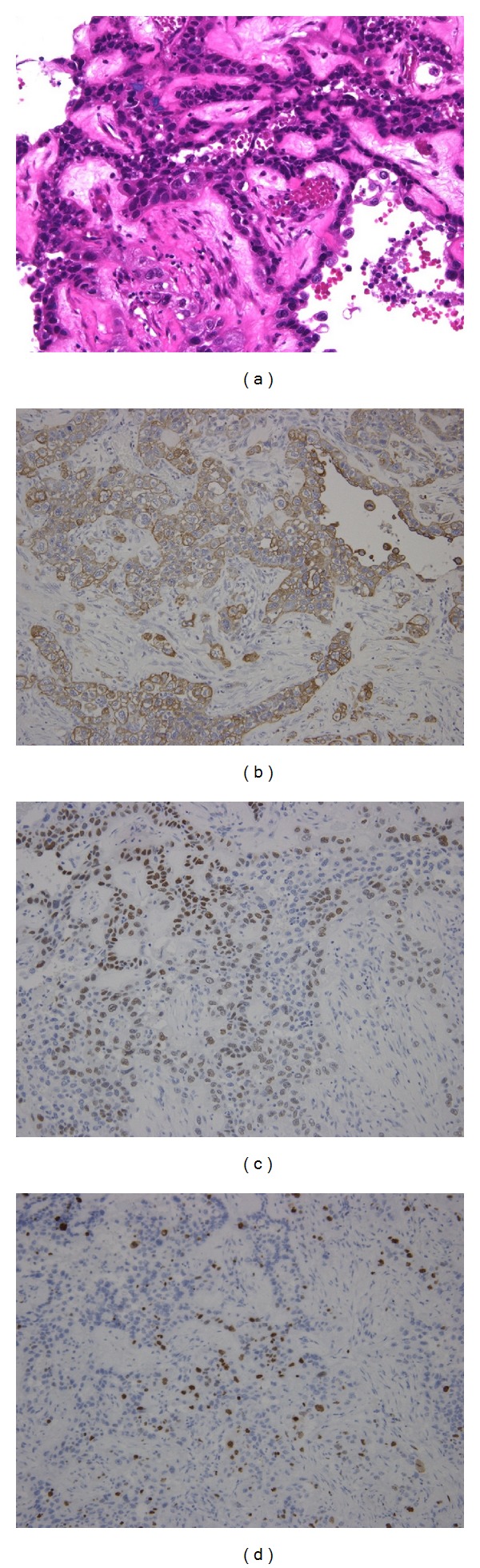
(a) Tubular structures with an inner layer of cuboidal epithelial cells and an outer layer of myoepithelial cells with clear cytoplasm. Mitoses were not conspicuous (hematoxylin-eosin, original magnification ×200). (b) Immunoreactivity in epithelial cells for cytokeratin (original magnification ×200). (c) Immunoreactivity in myoepithelial cells for p63 (original magnification ×200). (d) Immunoreactivity for the MIB-1 was about 10% (original magnification ×200).

An 81-year-old man was admitted to our hospital with a chief complaint of left leg motor weakness for one week. Neurological examination showed left hemiparesis (manual muscle test 4/5). Physical examination showed a subcutaneous tumor measuring 40 × 30 mm in the right frontal region. The tumor was not mobile and painless. Normal skin covered the tumor. There was no skin rash over the tumor. The patient had a medical history of prostate cancer at the age of 78 years. The prostate cancer had been totally resected with no signs of recurrence for 3 years. The patient had no smoking history. Laboratory studies showed the serum prostate-specific antigen level to be 1.763 (0–4) ng/mL. Skull radiography and computed tomography (CT) showed an osteolytic tumor in the right frontal bone (Figure 1(a)). Magnetic resonance imaging (MRI) showed a heterointense tumor with perifocal edema on both T1- and T2-weighted images, which was enhanced heterogeneously by gadolinium-diethylenetriaminepenta-acetic acid (Gd-DTPA). The tumor was attached to the wall of the superior sagittal sinus and dura of the right frontal lobe (Figures 1(b) and 1(c)). The preoperative workup included CT of the chest, abdomen, and pelvis, which demonstrated a right lung tumor with subcarinal lymph node swelling and pulmonary emphysema (Figure 1(d)). No other tumors, such as in the salivary gland and breast, were found. A wide scalp incision and craniotomy were performed with a 2-cm rim of bone over the superior sagittal sinus and tumor. The tumor was excised through the periosteum covering the bone (Figure 2(a)), and the margins were negative (Figure 2(b)). The tumor was attached to the wall of the superior sagittal sinus and surface of the brain. Subtotal resection was performed under a microscope. Dural plasty was conducted using a GORE-TEX surgical membrane. A titanium mesh plate was placed over the skull defect. Histologically, the tumor showed biphasic architecture, composed of glands and spindle cells. The glands had a double layer of cells. The inner cells were flattened or cuboidal with pink cytoplasm. The outer cells were round or polygonal and exhibited either clear or eosinophilic cytoplasm (Figure 3(a)). The inner cells showed immunoreactivity for cytokeratins (Figure 3(b)). The outer cells showed immunoreactivity for p63 (Figure 3(c)) and S-100, but not for TTF-1. The MIB-1 labeling index was approximately 10% in myoepithelial cells (Figure 3(d)). No necrosis was observed. Mitoses were not conspicuous. We diagnosed the tumor as EMC. Postoperatively, the patient's left hemiparesis improved completely, and no neurological deterioration manifested. A bronchoscopic biopsy was performed. The lung tumor was confirmed to be an EMC. Further examinations of the parotid and salivary glands were performed by an otolaryngologist, but no other tumors were detected. We concluded that pulmonary EMC had metastasized to the dura and skull. The MIB-1 index in primary lesion was approximately 10%. Whole brain radiation therapy (30 Gy) was then administered. For the lung tumor, best supportive care was provided for the will of the patient. 

## 3. Discussion

The tracheobronchial submucosal glands are the pulmonary counterpart of the salivary gland system. EMCs originating from bronchial submucosal glands are very rare. To date, approximately 20 cases have been reported in the English literature [1]. Doganay et al. reported a case with a review of 7 other cases in 2003. The patients were in the fifth to seventh decades of life, and 50% were male [2]. The clinical course of EMC is unknown. EMC is considered to be a low-grade tumor [3, 4], and treatment by complete surgical resection with negative margins is recommended [5, 6]. Some authors have pointed out its malignant potential, but little is known about the biological potential of EMC due to limited follow-up data [6–10]. However, even in a case with recurrence, local resection kept the patient asymptomatic for 4 years [1]. Intracranial metastasis of pulmonary EMC has not previously been reported, according to our search of the literature. Our case presentation seems to be the first of pulmonary EMC found after metastasis to the skull and dura hematogenously. Therefore, we felt it is important to report this case. Histologically, all lesions share the common features of having duct-like structures within a background of variable proportions of epithelial and myoepithelial cells [6]. The inner layer consists of small darker cuboidal epithelial cells (positive for cytokeratin, negative for S-100 and muscle-specific actin), while the outer layer is formed by clear myoepithelial cells (positive for S-100, muscle-specific actin, and P63 with focal weak positivity for cytokeratin) [3, 4, 6, 11]. As to the malignant potential of pulmonary EMC, samples obtained from the primary and metastatic regions in our case did not show prognostic factors such as mitoses or necrosis. Only immunoreactivity for the MIB-1 index in primary and metastatic lesions, up to 10%, suggested malignant potential. 

## 4. Conclusions

The skull and dura are possible sites of metastasis from pulmonary EMC. The MIB-1 index is a marker predicting malignant potential.
